# *trans*-Tetra­chloridobis(diphenyl­aceto­nitrile)platinum(IV)

**DOI:** 10.1107/S1600536809016535

**Published:** 2009-05-14

**Authors:** Nadezhda A. Bokach, Vadim Yu. Kukushkin, Matti Haukka

**Affiliations:** a Department of Chemistry, St Petersburg State University, Stary Petergof 198504, Russian Federation; b Institute of Macromolecular Compounds of the Russian Academy of Sciences, V. O. Bolshoi Pr. 31, 199004 St Petersburg, Russian Federation; c Department of Chemistry, University of Joensuu, PO Box 111, Joensuu FI-80101, Finland

## Abstract

In the title compound, [PtCl_4_(C_14_H_11_N)_2_], the Pt atom lies on an inversion center and has a distorted octa­hedral environment. The main geometric parameters are Pt—N = 1.960 (5) Å, and Pt—Cl = 2.3177 (12) and 2.3196 (12) Å. The N C bond is a typical triple bond [1.137 (7) Å]. The Pt—N C—C unit is almost linear, with Pt—N—C and N—C—C angles of 174.6 (4) and 177.1 (6)°, respectively.

## Related literature

For background literature, see: Kukushkin & Pombeiro (2002 ▶); Luzyanin *et al.* (2002 ▶); Pombeiro & Kukushkin (2004 ▶), For related structures, see: Allen *et al.* (1987 ▶); Eysel *et al.* (1983 ▶); Johansson *et al.* (1998 ▶); Kritzenberger *et al.* (1994 ▶); Orpen *et al.* (1989 ▶); Scollard *et al.* (2001 ▶); Svensson *et al.* (1995 ▶); Yagyu *et al.* (2002 ▶).
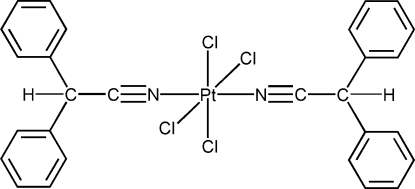


## Experimental

### 

#### Crystal data

[PtCl_4_(C_14_H_11_N)_2_]
*M* *_r_* = 723.37
Triclinic, 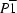

*a* = 5.7980 (3) Å
*b* = 10.8650 (6) Å
*c* = 11.2200 (7) Å
α = 92.236 (3)°
β = 101.601 (4)°
γ = 98.565 (4)°
*V* = 682.91 (7) Å^3^
*Z* = 1
Mo *K*α radiation
μ = 5.55 mm^−1^
*T* = 100 K
0.33 × 0.09 × 0.06 mm

#### Data collection

Nonius KappaCCD diffractometer
Absorption correction: multi-scan (*SADABS*; Sheldrick, 2008*a* ▶) *T*_min_ = 0.255, *T*_max_ = 0.717
13055 measured reflections
3096 independent reflections
3076 reflections with *I* > 2σ(*I*)
*R*_int_ = 0.048

#### Refinement

*R*[*F* ^2^ > 2σ(*F* ^2^)] = 0.038
*wR*(*F* ^2^) = 0.099
*S* = 1.09
3096 reflections
160 parameters
36 restraints
H-atom parameters constrained
Δρ_max_ = 4.19 e Å^−3^
Δρ_min_ = −2.02 e Å^−3^

### 

Data collection: *COLLECT* (Hooft, 2008 ▶); cell refinement: *DENZO*/*SCALEPACK* (Otwinowski & Minor, 1997 ▶); data reduction: *DENZO*/*SCALEPACK*; program(s) used to solve structure: *SIR2004* (Burla *et al.*, 2005 ▶); program(s) used to refine structure: *SHELXL97* (Sheldrick, 2008*b* ▶); molecular graphics: *DIAMOND* (Brandenburg, 2008 ▶); software used to prepare material for publication: *SHELXL97*.

## Supplementary Material

Crystal structure: contains datablocks I, global. DOI: 10.1107/S1600536809016535/pv2142sup1.cif

Structure factors: contains datablocks I. DOI: 10.1107/S1600536809016535/pv2142Isup2.hkl

Additional supplementary materials:  crystallographic information; 3D view; checkCIF report

## Figures and Tables

**Fig. 1.**
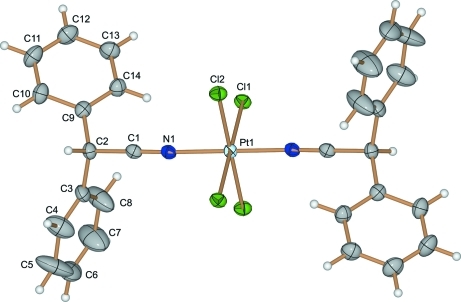
The molecular structure of the title molecule, with the atom-numbering scheme. Displacement ellipsoids are drawn at the 50% probability level.

## supplementary crystallographic information

### Comment

In the past decade, a Pt^IV^ center was recognized as one of the most efficient electrophilic activators of the C≡N bond in nitriles (Pombeiro & Kukushkin, 2004; Kukushkin & Pombeiro, 2002). Within the framework of our project focused on reactivity of metal-activated nitriles, a novel platinum(IV) complex, *i.e. trans*-[PtCl_4_(N≡CCHPh_2_)_2_], (I), was synthesized and characterized by single-crystal X-ray diffraction. It should be mentioned that only few structures of platinum(IV) nitrile complexes are known, e.g. (Yagyu *et al.*, 2002; Johansson *et al.*, 1998; Scollard *et al.*, 2001). Probably the small number of examples is related to the high reactivity of various (nitrile)Pt^IV^ species, where nitrile ligands are subject to facile nucleophilic attack even by weak nucleophiles or H_2_O in wet solvents.

The complex (I) crystallized in the centrosymmetrical P1 space group wherein the Pt atom lies on an inversion center and it has an octahedral environment and nitrile ligands have the mutual *trans* orientation (Fig. 1). The angles N1—Pt1—Cl2, N1—Pt1—Cl1, Cl2—Pt1—Cl1 are close to the ideal 90°. The Pt1—Cl bond distances (2.3177 (12) and 2.3196 (12) Å) are similar within 3σ with many other Pt—Cl bond lengths (2.323 (38) Å) in related Pt^IV^ complexes (Orpen *et al.*, 1989). The Pt1—N distances (1.960 (5) Å) are common for (nitrile)Pt complexes bearing two *trans*-coordinated nitriles, *e.g*. 1.943 (11)–1.978 (3) Å in Pt^II^ complexes (Eysel *et al.*, 1983; Kritzenberger *et al.*, 1994; Svensson *et al.* 1995).

The value of the N1≡C1 bond (1.137 (7) Å) is typical for the triple bonds in Pt^II^-coordinated (1.129 (9)–1.154 (18) Å in *trans*-[PtCl_2_(NCR)_2_] (Eysel *et al.*, 1983; Kritzenberger *et al.*, 1994; Svensson *et al.* 1995), in Pt^IV^-bound (1.09 (4)–1.157 (12) Å) (Yagyu *et al.*, 2002; Johansson *et al.*, 1998; Scollard *et al.*, 2001), and in uncomplexed (1.136 (10) Å (Allen *et al.*, 1987) nitriles. The value of the C1—C2 bond (1.469 (7) Å) agrees well with those reported for C*sp*–Csp^3^ single bonds (1.470 (13) Å) (Allen *et al.*, 1987). The Pt1/N1/C1/C2 moiety is almost linear with Pt1—N1≡C1 and N1≡C1—C2 angles of 174.6 (4) and 177.1 (6)°, correspondingly. The angle C3—C2—C9 (114.7 (5)°) is larger than 109° probably due to steric repulsion between two phenyl rings.

### Experimental

Diphenylacetonitrile (8.5 mg, 0.044 mmol; purchased from Aldrich) was added to a suspension of *trans*-[PtCl_4_(EtCN)_2_] (9.7 mg, 0.022 mmol) (Luzyanin *et al.*, 2002) in CDCl_3_ (1 ml) and the reaction mixture was left to stand for 2 d at 323 K in an NMR tube, whereupon orange–yellow crystals were formed on walls of the tube. The crystals were mechanically separated.

### Refinement

The phenyl ring C3–C8 was slightly disordered. However, no disordered model was used in the final refined but the C atoms on phenyl ring C3–C8 were restrained with effective standard deviation 0.1 so that its *U_ij_* components approximate to isotropic behavior. All H atoms were positioned geometrically and constrained to ride on their parent atoms, with C—H = 0.95 and 1.00 Å, for methine and aryl H atoms, respectively, and *U*_iso_ = 1.2*U*_eq_(parent atom). The residual electron density in the final difference map could be attributed to insufficient absorption correction as well as twinning, which could not be corrected.

### Crystal data

**Table:**

[PtCl_4_(C_14_H_11_N)_2_]	*Z* = 1
*M**_r_* = 723.37	*F*(000) = 350
Triclinic, *P*1	*D*_x_ = 1.759 Mg m^−^^3^
Hall symbol: -P 1	Mo *K*α radiation, λ = 0.71073 Å
*a* = 5.7980 (3) Å	Cell parameters from 30861 reflections
*b* = 10.8650 (6) Å	θ = 1.0–27.5°
*c* = 11.2200 (7) Å	µ = 5.55 mm^−^^1^
α = 92.236 (3)°	*T* = 100 K
β = 101.601 (4)°	Needle, yellow
γ = 98.565 (4)°	0.33 × 0.09 × 0.06 mm
*V* = 682.91 (7) Å^3^	

### Data collection

**Table:**

Nonius KappaCCD diffractometer	3096 independent reflections
Radiation source: fine-focus sealed tube	3076 reflections with *I* > 2σ(*I*)
horizontally mounted graphite crystal	*R*_int_ = 0.048
Detector resolution: 9 pixels mm^-1^	θ_max_ = 27.4°, θ_min_ = 1.9°
φ scans and ω scans with κ offset	*h* = −6→7
Absorption correction: multi-scan (*SADABS*; Sheldrick, 2008a)	*k* = −13→14
*T*_min_ = 0.255, *T*_max_ = 0.717	*l* = −14→14
13055 measured reflections	

### Refinement

**Table:**

Refinement on *F*^2^	Primary atom site location: structure-invariant direct methods
Least-squares matrix: full	Secondary atom site location: difference Fourier map
*R*[*F*^2^ > 2σ(*F*^2^)] = 0.038	Hydrogen site location: inferred from neighbouring sites
*wR*(*F*^2^) = 0.099	H-atom parameters constrained
*S* = 1.09	*w* = 1/[σ^2^(*F*_o_^2^) + (0.0722*P*)^2^ + 0.3244*P*] where *P* = (*F*_o_^2^ + 2*F*_c_^2^)/3
3096 reflections	(Δ/σ)_max_ < 0.001
160 parameters	Δρ_max_ = 4.19 e Å^−^^3^
36 restraints	Δρ_min_ = −2.02 e Å^−^^3^

### Special details

**Table:**

Experimental. IR spectrum in KBr, selected bonds, cm^-1^: 2340 s ν(C≡N). ^1^H NMR spectrum in CDCl_3_, δ: 5.85 (s, 1H, CH), 7.42 (m, 10H, Ph).
Geometry. All e.s.d.'s (except the e.s.d. in the dihedral angle between two l.s. planes) are estimated using the full covariance matrix. The cell e.s.d.'s are taken into account individually in the estimation of e.s.d.'s in distances, angles and torsion angles; correlations between e.s.d.'s in cell parameters are only used when they are defined by crystal symmetry. An approximate (isotropic) treatment of cell e.s.d.'s is used for estimating e.s.d.'s involving l.s. planes.
Refinement. Refinement of *F*^2^ against ALL reflections. The weighted *R*-factor *wR* and goodness of fit *S* are based on *F*^2^, conventional *R*-factors *R* are based on *F*, with *F* set to zero for negative *F*^2^. The threshold expression of *F*^2^ > σ(*F*^2^) is used only for calculating *R*-factors(gt) *etc*. and is not relevant to the choice of reflections for refinement. *R*-factors based on *F*^2^ are statistically about twice as large as those based on *F*, and *R*- factors based on ALL data will be even larger.

### Fractional atomic coordinates and isotropic or equivalent isotropic displacement parameters (Å^2^)

**Table:**

	*x*	*y*	*z*	*U*_iso_*/*U*_eq_	
Pt1	0.0000	0.0000	0.0000	0.01907 (11)	
Cl1	−0.1642 (2)	0.18164 (11)	−0.02653 (11)	0.0242 (3)	
Cl2	0.1559 (2)	0.03117 (12)	−0.17314 (11)	0.0259 (3)	
N1	0.2869 (8)	0.0989 (4)	0.1023 (4)	0.0219 (9)	
C1	0.4426 (9)	0.1634 (5)	0.1627 (5)	0.0223 (10)	
C2	0.6350 (10)	0.2492 (5)	0.2438 (5)	0.0244 (10)	
H2	0.7905	0.2282	0.2306	0.029*	
C3	0.6198 (10)	0.2260 (6)	0.3764 (5)	0.0304 (12)	
C4	0.7693 (19)	0.1559 (9)	0.4421 (7)	0.060 (2)	
H4	0.8855	0.1233	0.4068	0.072*	
C5	0.751 (3)	0.1322 (10)	0.5624 (8)	0.083 (4)	
H5	0.8513	0.0808	0.6067	0.100*	
C6	0.5929 (16)	0.1807 (9)	0.6164 (7)	0.058 (2)	
H6	0.5877	0.1677	0.6992	0.069*	
C7	0.442 (2)	0.2487 (14)	0.5495 (9)	0.084 (3)	
H7	0.3263	0.2819	0.5849	0.101*	
C8	0.4554 (18)	0.2700 (12)	0.4286 (8)	0.071 (3)	
H8	0.3468	0.3164	0.3825	0.085*	
C9	0.6217 (10)	0.3826 (5)	0.2100 (5)	0.0253 (11)	
C10	0.8298 (11)	0.4709 (6)	0.2383 (6)	0.0335 (13)	
H10	0.9758	0.4464	0.2767	0.040*	
C11	0.8249 (13)	0.5941 (6)	0.2106 (7)	0.0415 (15)	
H11	0.9671	0.6535	0.2298	0.050*	
C12	0.6130 (13)	0.6299 (6)	0.1552 (7)	0.0396 (14)	
H12	0.6094	0.7139	0.1355	0.047*	
C13	0.4058 (12)	0.5434 (6)	0.1285 (6)	0.0352 (13)	
H13	0.2597	0.5691	0.0919	0.042*	
C14	0.4085 (11)	0.4201 (5)	0.1543 (5)	0.0297 (12)	
H14	0.2656	0.3612	0.1342	0.036*	

### Atomic displacement parameters (Å^2^)

**Table:**

	*U*^11^	*U*^22^	*U*^33^	*U*^12^	*U*^13^	*U*^23^
Pt1	0.02018 (16)	0.01738 (16)	0.01892 (16)	0.00369 (10)	0.00211 (10)	0.00008 (10)
Cl1	0.0285 (6)	0.0191 (6)	0.0254 (6)	0.0078 (5)	0.0035 (5)	0.0017 (5)
Cl2	0.0321 (7)	0.0243 (6)	0.0224 (6)	0.0052 (5)	0.0078 (5)	0.0018 (5)
N1	0.022 (2)	0.023 (2)	0.022 (2)	0.0071 (17)	0.0047 (17)	0.0036 (17)
C1	0.025 (3)	0.021 (2)	0.023 (2)	0.008 (2)	0.005 (2)	0.002 (2)
C2	0.023 (2)	0.024 (3)	0.024 (2)	0.002 (2)	0.002 (2)	−0.002 (2)
C3	0.027 (3)	0.037 (3)	0.023 (2)	0.001 (2)	−0.001 (2)	0.001 (2)
C4	0.084 (5)	0.059 (4)	0.041 (4)	0.035 (4)	0.007 (3)	0.002 (3)
C5	0.150 (11)	0.072 (7)	0.033 (4)	0.057 (7)	0.002 (5)	0.014 (4)
C6	0.066 (5)	0.072 (5)	0.027 (3)	−0.008 (4)	0.001 (3)	0.007 (3)
C7	0.078 (6)	0.139 (8)	0.046 (4)	0.037 (6)	0.024 (4)	0.019 (5)
C8	0.063 (5)	0.123 (7)	0.039 (4)	0.046 (5)	0.013 (3)	0.019 (4)
C9	0.028 (3)	0.024 (3)	0.025 (2)	0.003 (2)	0.008 (2)	−0.002 (2)
C10	0.030 (3)	0.029 (3)	0.039 (3)	−0.001 (2)	0.007 (2)	−0.006 (2)
C11	0.040 (4)	0.028 (3)	0.055 (4)	−0.004 (3)	0.015 (3)	−0.001 (3)
C12	0.046 (4)	0.025 (3)	0.049 (4)	0.004 (3)	0.014 (3)	0.001 (3)
C13	0.040 (3)	0.028 (3)	0.040 (3)	0.011 (2)	0.008 (3)	0.004 (2)
C14	0.030 (3)	0.024 (3)	0.034 (3)	0.003 (2)	0.005 (2)	−0.001 (2)

### Geometric parameters (Å, °)

**Table:**

Pt1—N1^i^	1.960 (5)	C6—C7	1.360 (15)
Pt1—N1	1.960 (5)	C6—H6	0.9500
Pt1—Cl2	2.3177 (12)	C7—C8	1.400 (12)
Pt1—Cl2^i^	2.3178 (12)	C7—H7	0.9500
Pt1—Cl1	2.3196 (12)	C8—H8	0.9500
Pt1—Cl1^i^	2.3196 (12)	C9—C14	1.394 (8)
N1—C1	1.137 (7)	C9—C10	1.398 (8)
C1—C2	1.469 (7)	C10—C11	1.389 (9)
C2—C9	1.522 (8)	C10—H10	0.9500
C2—C3	1.536 (8)	C11—C12	1.379 (10)
C2—H2	1.0000	C11—H11	0.9500
C3—C8	1.348 (11)	C12—C13	1.383 (9)
C3—C4	1.363 (10)	C12—H12	0.9500
C4—C5	1.406 (13)	C13—C14	1.383 (9)
C4—H4	0.9500	C13—H13	0.9500
C5—C6	1.350 (15)	C14—H14	0.9500
C5—H5	0.9500		
			
N1^i^—Pt1—N1	180.0	C6—C5—H5	119.2
N1^i^—Pt1—Cl2	88.94 (13)	C4—C5—H5	119.2
N1—Pt1—Cl2	91.06 (13)	C5—C6—C7	118.4 (8)
N1^i^—Pt1—Cl2^i^	91.06 (13)	C5—C6—H6	120.8
N1—Pt1—Cl2^i^	88.94 (13)	C7—C6—H6	120.8
Cl2—Pt1—Cl2^i^	180.0	C6—C7—C8	120.2 (10)
N1^i^—Pt1—Cl1	91.31 (13)	C6—C7—H7	119.9
N1—Pt1—Cl1	88.69 (13)	C8—C7—H7	119.9
Cl2—Pt1—Cl1	89.95 (5)	C3—C8—C7	121.4 (9)
Cl2^i^—Pt1—Cl1	90.05 (5)	C3—C8—H8	119.3
N1^i^—Pt1—Cl1^i^	88.69 (13)	C7—C8—H8	119.3
N1—Pt1—Cl1^i^	91.31 (13)	C14—C9—C10	119.1 (6)
Cl2—Pt1—Cl1^i^	90.05 (5)	C14—C9—C2	122.2 (5)
Cl2^i^—Pt1—Cl1^i^	89.95 (5)	C10—C9—C2	118.7 (5)
Cl1—Pt1—Cl1^i^	180.0	C11—C10—C9	120.5 (6)
C1—N1—Pt1	174.6 (4)	C11—C10—H10	119.7
N1—C1—C2	177.1 (6)	C9—C10—H10	119.7
C1—C2—C9	109.6 (4)	C12—C11—C10	119.8 (6)
C1—C2—C3	108.4 (5)	C12—C11—H11	120.1
C9—C2—C3	114.7 (5)	C10—C11—H11	120.1
C1—C2—H2	107.9	C11—C12—C13	120.0 (6)
C9—C2—H2	107.9	C11—C12—H12	120.0
C3—C2—H2	107.9	C13—C12—H12	120.0
C8—C3—C4	118.8 (7)	C12—C13—C14	120.8 (6)
C8—C3—C2	121.4 (6)	C12—C13—H13	119.6
C4—C3—C2	119.8 (6)	C14—C13—H13	119.6
C3—C4—C5	119.6 (9)	C13—C14—C9	119.8 (6)
C3—C4—H4	120.2	C13—C14—H14	120.1
C5—C4—H4	120.2	C9—C14—H14	120.1
C6—C5—C4	121.6 (8)		

Symmetry codes: (i) −*x*, −*y*, −*z*.

## References

[bb1] Allen, F. H., Kennard, O., Watson, D. G., Brammer, L., Orpen, A. G. & Taylor, R. (1987). *J. Chem. Soc. Perkin Trans. 2*, pp. S1–19.

[bb2] Brandenburg, K. (2008). *DIAMOND* Crystal Impact GbR, Bonn, Germany.

[bb3] Burla, M. C., Caliandro, R., Camalli, M., Carrozzini, B., Cascarano, G. L., De Caro, L., Giacovazzo, C., Polidori, G. & Spagna, R. (2005). *J. Appl. Cryst.* **38**, 381–388.

[bb4] Eysel, H. H., Guggolz, E., Kopp, M. & Ziegler, M. L. (1983). *Z. Anorg. Allg. Chem* **499**, 31–43.

[bb5] Hooft, R. (2008). *COLLECT* Bruker AXS, Delft, The Netherlands.

[bb6] Johansson, L., Ryan, O. B., Romming, C. & Tilset, M. (1998). *Organometallics*, **17**, 3957–3966.

[bb7] Kritzenberger, J., Yersin, H., Range, K.-J. & Zabel, M. Z. (1994). *Z. Naturforsch. Teil B*, **49**, 297–300.

[bb8] Kukushkin, V. Yu. & Pombeiro, A. J. L. (2002). *Chem. Rev.* **102**, 1771–1802. 10.1021/cr0103266 11996549

[bb9] Luzyanin, K. V., Haukka, M., Bokach, N. A., Kuznetsov, M. L., Kukushkin, V. Y. & Pombeiro, A. J. L. (2002). *J. Chem. Soc. Dalton Trans.* pp. 1882–1887.

[bb11] Orpen, A. G., Brammer, L., Allen, F. H., Kennard, O., Watson, D. G. & Taylor, R. (1989). *J. Chem. Soc. Dalton Trans.* pp. S1–S83.

[bb12] Otwinowski, Z. & Minor, W. (1997). *Methods in Enzymology*, Vol. 276, *Macromolecular Crystallography*, Part A, edited by C. W. Carter & R. M. Sweet, pp. 307–326. New York: Academic Press.

[bb13] Pombeiro, A. J. L. & Kukushkin, V. Y. (2004). *Comprehensive Coordination Chemistry II*, Vol. 1, edited by A. B. P. Lever, pp. 639–660. Elsevier.

[bb14] Scollard, J. D., Day, M., Labinger, J. A. & Bercaw, J. E. (2001). *Helv. Chim. Acta*, **84**, 3247–3268.

[bb15] Sheldrick, G. M. (2008*a*). *SADABS.* Bruker AXS, Wisconsin, USA.

[bb16] Sheldrick, G. M. (2008*b*). *Acta Cryst.* A**64**, 112–122. 10.1107/S0108767307043930 18156677

[bb17] Svensson, P., Lövqvist, K., Kukushkin, V. Y. & Oskarsson, Å. (1995). *Acta Chem. Scand.* **49**, 72–75.

[bb18] Yagyu, T., Suzaki, Y. & Osakada, K. (2002). *Organometallics*, **21**, 2088–2094.

